# Lineage divergence detected in the malaria vector *Anopheles marajoara *(Diptera: Culicidae) in Amazonian Brazil

**DOI:** 10.1186/1475-2875-9-271

**Published:** 2010-10-07

**Authors:** Sascha N McKeon, Margaret A Lehr, Richard C Wilkerson, John F Ruiz, Maria A Sallum, Jose BP Lima, Marinete M Povoa, Jan E Conn

**Affiliations:** 1 Department of Biomedical Sciences, School of Public Health, State University of New York-Albany, Empire State Plaza, Albany, NY 12201 USA; 2 Department of Biology, 120A Marsh Life Sciences Building, University of Vermont, Burlington, VT 05405, USA; 3 Department of Entomology, Walter Reed Army Institute of Research, 503 Robert Grant Avenue, Silver Spring, MD 20910 USA; 4 Department of Epidemiology, Universidade de Sao Paulo, 715 - Cerqueira Cesar 01246-904 Sao Paulo, Brazil; 5 Laboratório de Fisiologia e Controle de Artropodes Vetores, IOC-Fiocruz, 4365 - Manguinhos, Rio de Janeiro, Brazil; 6 Laboratório de Pesquisas Básicas em Malaria, Instituto Evandro Chagas, Secretaria de Vigilância em Saúde/MS, Br 316, Km 7, s/n, CEP 67.030-000, Ananindeua, Para, Brazil; 7 The Wadsworth Center, Griffin Laboratory, New York State Department of Health, 5668 State Farm Road, Slingerlands, NY 12159, USA

## Abstract

**Background:**

Cryptic species complexes are common among anophelines. Previous phylogenetic analysis based on the complete mtDNA *COI *gene sequences detected paraphyly in the Neotropical malaria vector *Anopheles marajoara*. The "Folmer region" detects a single taxon using a 3% divergence threshold.

**Methods:**

To test the paraphyletic hypothesis and examine the utility of the Folmer region, genealogical trees based on a concatenated (*white *+ 3' *COI *sequences) dataset and pairwise differentiation of *COI *fragments were examined. The population structure and demographic history were based on partial *COI *sequences for 294 individuals from 14 localities in Amazonian Brazil. 109 individuals from 12 localities were sequenced for the nDNA *white *gene, and 57 individuals from 11 localities were sequenced for the ribosomal DNA (rDNA) internal transcribed spacer 2 (ITS2).

**Results:**

Distinct *A. marajoara *lineages were detected by combined genealogical analysis and were also supported among *COI *haplotypes using a median joining network and AMOVA, with time since divergence during the Pleistocene (<100,000 ya). *COI *sequences at the 3' end were more variable, demonstrating significant pairwise differentiation (3.82%) compared to the more moderate 2.92% detected by the Folmer region. Lineage 1 was present in all localities, whereas lineage 2 was restricted mainly to the west. Mismatch distributions for both lineages were bimodal, likely due to multiple colonization events and spatial expansion (~798 - 81,045 ya). There appears to be gene flow within, not between lineages, and a partial barrier was detected near Rio Jari in Amapá state, separating western and eastern populations. In contrast, both nDNA data sets (*white *gene sequences with or without the retention of the 4th intron, and ITS2 sequences and length) detected a single *A. marajoara *lineage.

**Conclusions:**

Strong support for combined data with significant differentiation detected in the *COI *and absent in the nDNA suggest that the divergence is recent, and detectable only by the faster evolving mtDNA. A within subgenus threshold of >2% may be more appropriate among sister taxa in cryptic anopheline complexes than the standard 3%. Differences in demographic history and climatic changes may have contributed to mtDNA lineage divergence in *A. marajoara*.

## Background

About 1.8 million species are known on Earth, including more than 1 million insects, 250,000 higher plants and 69,000 fungi [1]. The Amazon comprises much of this biodiversity and is considered the largest gene reserve in the world, with an estimated 14% of all plant and animal species within its boundaries [2]. Because speciation is not always accompanied by morphological change [3], the true number of biological species is likely to be greater than current estimates [4]. Genetic analysis plays an increasingly important role in identifying changes in population structure and elucidating taxonomic status and phylogenetic relationships.

Genetically divergent but morphologically cryptic species have been described in many aquatic organisms [5], birds [6,7] and insects [8,9], particularly among mosquitoes in the Dipteran genus *Anopheles *[10-12]. The Neotropical Albitarsis Complex contains at least six species, only some of which are documented malaria vectors: *Anopheles marajoara*, a local and regionally important vector in lowland rainforest [13-15], *Anopheles janconnae *(previously *Anopheles albitarsis E*) implicated in local transmission in Amazonian savannah [16]; and *Anopheles deaneorum*, which appears to consist of multiple species [17], and is a potential vector, as demonstrated by comparative susceptibility laboratory studies [18,19].

Various methods have been used to investigate species delimitation and identifications in the Albitarsis Complex [11,20,21], culminating, most recently, in the recognition of six species [22], but see Bourke *et al *[17]. Genealogical analyses of complete mtDNA *COI *(Cytochrome oxidase I) sequences found that *A. marajoara *is paraphyletic, and may consist of at least two phylogenetic species [10] or lineages [3,23], one of which may be *A. janconnae*.

The mitochondrial genome has been used extensively in studies of molecular evolution [24] and the *COI *gene has resolved evolutionary relationships among closely related species for a wide range of taxa [25,26], including insects [27,28] and cryptic species complexes [29,30]. The Folmer region, 648-bp at the 5' end of the *COI *mitochondrial gene, has emerged as the standard barcode region [31,32]. Interspecific divergence within insects almost always exceeds 3% and this value has been used as a speciation threshold [33]. The true test of DNA barcode precision would include comparisons with sister species [34]. The utility of DNA barcoding among insects is still being debated, because of success in revealing cryptic species [31,35,36] on one hand, and the inability to reliably detect species boundaries on the other [36-38]. An estimated 20% failure rate has been noted at the species level due to non-monophyly [39], which increases among insects due to overlapping ranges of intra- and interspecific sequence divergences [40]. Together these findings suggest that the threshold level could be set lower than 3% to minimize false negatives [36].

Accurate morphological identification of the adult females of species within the Albitarsis Complex is virtually impossible, and the ITS2 has become a recognized molecular tool for identification [21,41,42]. ITS2 variation is low within a species due to homogenization and fixation while the overall fragment length is generally variable between species [20,43-45]. As such it can usually resolve phylogenetic relationships at different taxonomic levels, and detect recently diverged taxa such as sibling species of mosquitoes [46].

Members of a species are rarely distributed homogeneously in space, and population subdivision can occur in response to geographical boundaries, social behaviour and genetic variation [47]. Patterns in biological sequence data that arise from ancestry can be useful in determining the structure and boundaries of a given species [48]. The objective of this study was to test the hypothesis of paraphyly [10] using a combined data set (*white *gene + *COI*) and to evaluate the population structure of *A. marajoara *to address the following questions: 1) is the proposed paraphyletic status supported; 2) what is the level of genetic differentiation between populations; 3) can lineages of *A. marajoara *be distinguished by the Barcode of Life (BOLD) 3% species threshold; and 4) can genetic differentiation be explained by demographic phenomena, geographic boundaries and (or) natural selection.

## Methods

### Mosquito collection

Adult female mosquitoes from seven localities spanning roughly 890 kilometers of a transect along the Amazon River were collected using Shannon traps adjacent to breeding habitats between Amazonas state near Urucara along the Amazon river and the tributary of Rio Paru entering the Amazon river. They were also collected from Itaituba, south of the Amazon River, Pará State, in 2005 (collection protocol approved by the New York State Department of Health IRB and Brazilian Instituto Evandro Chagas, Belém, Pará state Ethical Committee). Mosquitoes were identified morphologically using the key of Deane et al. [49] as *A. albitarsis *s.l. Previously extracted specimens collected between 1995 and 2001, using a human landing catch protocol approved by the University of Vermont and the Brazilian Instituto Evandro Chagas, Belém, Pará state Ethical Committee, from seven localities in northeastern Pará and Amapá states of Brazil and from Itaituba, south of the Amazon River in Pará State, were also included [50]. Genomic DNA was extracted using the Puregene DNA isolation kit (Gentra Systems) and maintained at Griffin Laboratory at -80°C. Ten to 27 mosquitoes per site were selected for DNA amplification and sequencing of the *COI *(Table 1). A subset of four to 15 samples for the *white *gene and four to seven samples for ITS2 were amplified and sequenced (Table 1).

**Table 1:** *Anopheles albitarsis *s.l. collection information

				Sample Size		
				
Site	Locality	State	Coordinates	*COI*	*white*	ITS2
1	Urucara	Amazonas	S2.32 W57.46	25	15	7

2	Paratins	Amazonas	S2.37 W56.39	25	7	6

3	Campina	Pará	S2.52 W55.28	25	12	5

4	Itaituba	Pará	S4.10 W55.50	27	12	4

5	Santarém	Pará	S2.26 W54.45	25	10	6

6	Monte Alegre	Pará	S2.01 W54.05	25	9	6

7	Uruará	Pará	S2.08 W53.38	25	9	6

8	Rio Paru	Pará	S1.28 W52.44	25	13	7

9	Serra do Navio	Amapá	N0.53 W52.00	14	0	0

10	Santana	Amapá	S0.20 W51.11	18	9	2

11	Macapá	Amapá	N0.20 W51.30	21	4	2

12	Tartarugalzinho	Amapá	N1.19 W50.57	15	4	0

13	Amapá	Amapá	N2.10 W50.54	10	0	0

14	Salvaterra	Pará	S0.46 W48.31	14	5	2

**Total**				**294**	**107**	**53**

### Amplification and sequencing

A 1200-bp fragment of the *COI *gene was amplified using the forward primer UEA3 and the reverse primer UEA10 [28]. An 822-bp fragment from the 3' end using PCR primers: 2195D (5'-TGATTYTTTGGTCATCCNGAAGT-3'; a modification of C1-J-2195 for amplification in Collembola [51]) and FLY10A (5'-AATGCACTAATCTGCCATATTAG-3'; a modification of TL2-N-3014 for amplification in Simuliid flies [52]) was previously amplified for the northeastern collection samples [50]. Individual PCR reactions were preformed using Ready-To-Go-PCR bead (Amersham Pharmacia/Biotech, NJ, USA) and run on a PTC 100 or 200 series thermal cycler (Biorad, Inc.), or a PTC-100 (MJ Research, Inc.), using the conditions stipulated in Mirabello and Conn [53]. The Applied Genomics Technology Core (Wadsworth Center) carried out the sequencing. The forward and reverse *COI *sequences were aligned using Sequencher 3.0 (Gene Codes Corps, MI, USA), grouped together by sight and trimmed in PAUP*, version 4.0 [54]. The complete overlap of both *COI *primer sets created a 488-bp fragment (Additional file 1).

An 800-bp fragment of the *white *gene was amplified using the W2R and WF primers, with the PCR conditions as reported in Mirabello and Conn [55]. A 500-bp fragment of the ribosomal ITS2 was amplified using the primers 18S and 28S with the parameters in Li and Wilkerson [20]. The PCR products were cleaned, sequenced and aligned creating a 496-bp fragment of the *white *gene and 361-bp of the ITS2 with complete forward and reverse overlap for each marker. Unique sequences for all markers are available in GenBank [GenBank: HQ026025-HQ026113].

### Phylogenetic relationship

All *A. albitarsis *s.l. sequences were identified to species by multiple gene (*COI*, *white*, ITS2) comparisons to those deposited in GenBank: *A. albitarsis *[DQ076207/DQ076208; AY956299/AY956300; AF462386/AF462387], *A. oryzalimnetes*, formerly *A. albitarsis *B [DQ076210-DQ076215; AY956297/AY956298; U92333], *A. marajoara *[DQ076216/DQ076217-DQ076221/DQ076225; AY956295/AY956296; U92334], *A. deaneorum *[DQ076226/DQ076229; AY956301/AY956302; AF461751/AF461752], *A. janconnae*, [DQ076231/DQ076232] and *A. albitarsis *F [DQ228314/DQ228315].

Genealogical trees were estimated using the concatenated (*white *+ *COI*) data set. ITS2 sequences were excluded from these analyses because of relatively limited sample size. Maximum Parsimony trees were generated in PAUP*, with one hundred replicates of a heuristic search performed with an initial random stepwise addition of sequences and TBR branch swapping. Branch support was estimated from 1,000 replicates of a bootstrap search. Bayesian inference (BI) analysis was performed with Mr. Bayes version 3.1 [56,57], partitioned by gene, using the model of nucleotide substitution (TPM3uf+G and TIM1+I) that best fit the *white *gene and *COI *respectively, determined with jModelTest [58,59]. The settings were two simultaneous, independent runs of the Markov Chain Monte Carlo (MCMC) for 4 million generations, sampling every 1,000 generations with a 'burnin' of 25%. The outgroup *A. albimanus *in the Albimanus Section of *Nyssorhynchus *(unpublished sequence) was chosen based upon its phylogenetic position in Sallum *et al *[60]. Estimates of time to coalescence were calculated for the *COI *fragment only and compared using *θ*_*S *_values [61] and BEAST [62].

### Genetic variation

Genetic structure of two lineages was examined by analysis of molecular variance (AMOVA), a method of estimating population variance directly from molecular data, using Arlequin version 3.1.1 [63]. In addition, spatial analysis of molecular variance (SAMOVA), version 1.0 [64] was used to cluster the 488-bp *COI *sequence data into genetically and geographically homogeneous populations. SAMOVA generates *F *statistics (*F*_*SC*_, *F*_*ST*_, *F*_*CT*_) using the AMOVA approach, into *K *groups to maximize the between group variation. SAMOVA estimates were computed for *K *= 2-13 with 1,000 simulated annealing steps from each of 100 sets of initial starting conditions.

A comparison of the pairwise divergences calculated by DnaSP between *COI *lineages using the 648-bp 5' end and the 488-bp 3' end tested the utility of the Folmer region and the 3% species threshold using 10 to 13 individuals from each lineage, three closely related taxa and an outgroup (*Anopheles darlingi*). *Anopheles darlingi *is more appropriate than *A. albimanus *for sister taxa comparisons as both it, and *A. marajoara*, are in the Argyritarsis Section [65]. A species screening threshold (SST) [66] may be more appropriate and can be calculated as 10× the mean intraspecific variation for a group [67]. The standard sequence threshold was calculated and examined for *Nyssorhynchus *using 4-58 archived GenBank sequences for known species.

### Population structure and demographic history

A statistical parsimony network estimated genealogical relationships among *COI *haplotypes with a 93% sequence identity and 95% identity for each of the nuclear markers using TCS 1.13 [68]. Homoplasy in all networks was resolved using the algorithm estimation rules in Crandall and Templeton [69].

The differentiation and polymorphism statistics for *COI *sequences by species or lineage and locality were computed in DnaSP, Version 4.0 [70], and the hypothesis of strict neutrality was examined using the statistics *D*_*T *_[71], *D *and *F *[72], and *R*_*2 *_[73] which are based on the frequencies of segregating sites, and Fu's *F*_*S *_[74], based on the haplotype distribution. Tajima's *D *and both Fu and Li's D* and F* are the most effective tests to detect background selection, whereas Fu's *F*_*S *_and *R*_*2 *_are among the most powerful tests to detect population expansion [73]. All neutrality tests were calculated using DnaSP, Version 4.0 or MEGA version 3.1 [75].

The mismatch distribution (simulated in Arlequin) is a frequency distribution of the observed number of pairwise nucleotide differences. The shape of the distribution is highly informative and able to differentiate between a population expansion and equilibrium [76], whereas the smoothness (raggedness statistic) distinguishes the fit of the empirical data to the model [77]. Statistically significant differences between observed and simulated distributions were evaluated with the sum of square deviations (SSD) to reject the hypothesis of demographic expansion [78].

## Results

### Phylogenetic relationship

**Figure 1:**
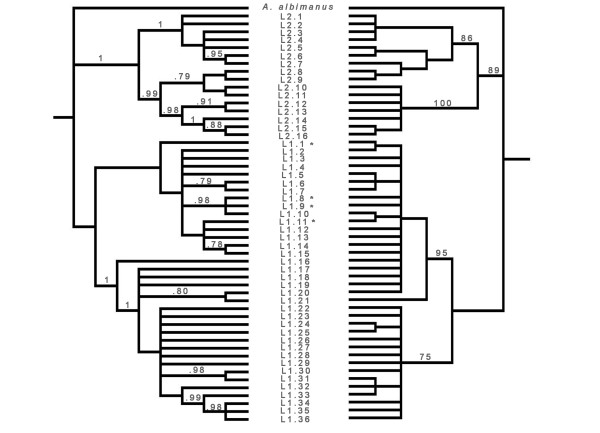
**Genealogical trees based on 52 haplotypes from 895-bp sequenced from 107 specimens of *A. marajoara *s.l**. Bayesian Inference (BI) and Maximum parsimony (MP) trees based on the combined *white *+ *COI *dataset. Branch support was estimated from 1,000 replicates of a bootstrap search (right tree) and posterior probability (left tree). *Anopheles albimanus*, was the out group. Lineage 2 haplotypes are indicated as L2.1-L2.16 and lineage 1 haplotypes as L1.1-L1.36. * Denotes samples from Marajo island, ~56 km from the *A. marajoara *type locality.

**Figure 2:**
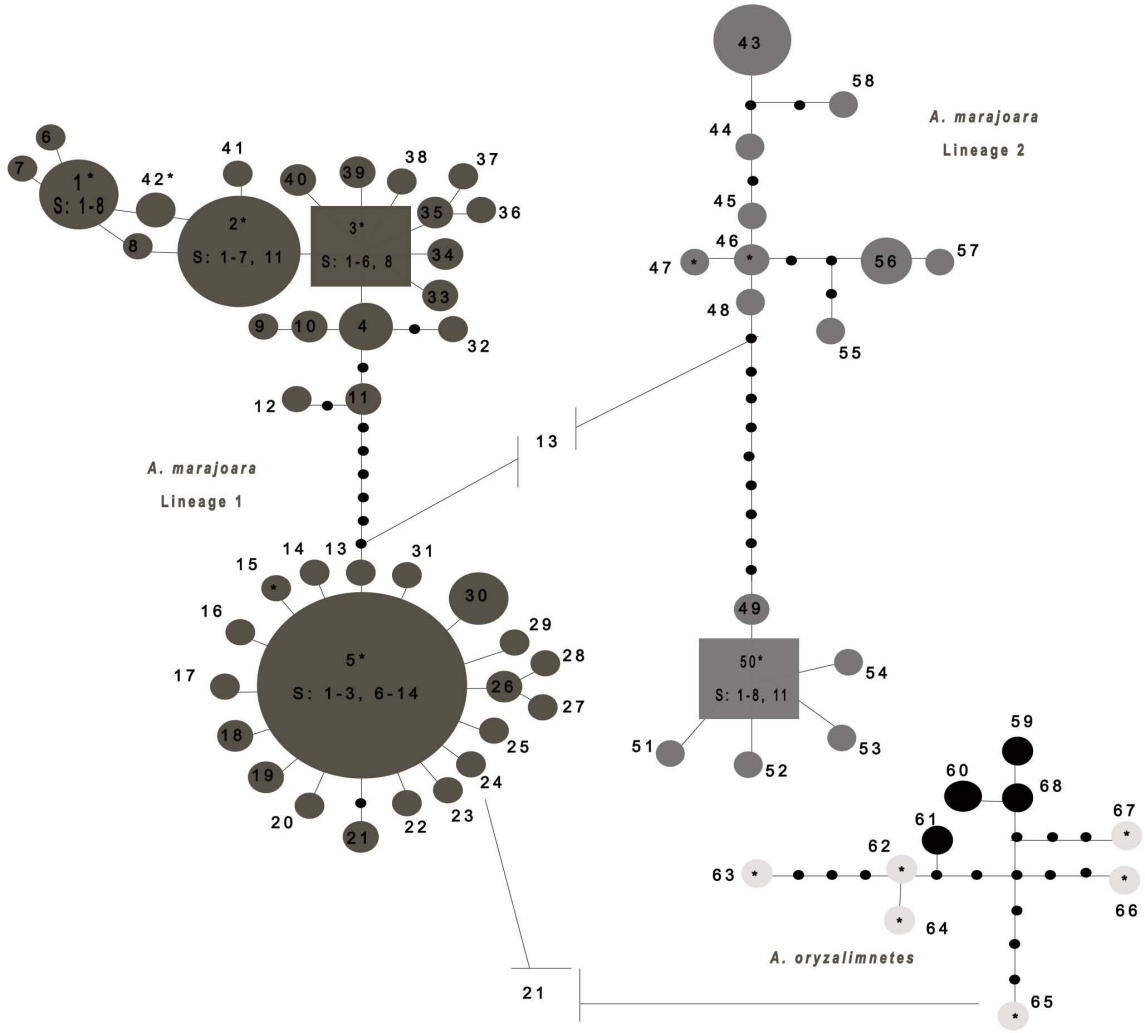
**Parsimony-based haplotype networks of 68 haplotypes from 488-bp of the *COI *gene sequenced from 294 specimens of *A. marajoara *s.l**. Statistical parsimony network of the *COI *haplotypes (see Additional file 2) from the 14 Amazonian localities based on a 488-bp fragment and 93% sequence similarity. Dark grey represents *A. marajoara *lineage 1; light grey, lineage 2; and black indicates *A. oryzalimnetes*. Each line represents a single mutational event; black dots represent unobserved, possibly extinct, haplotypes. Haplotype numbers are indicated in bold, * denotes the inclusion of known GenBank sequences identical to haplotypes 1, 2, 3, 5, 42, 46, 47, and 50 identified as *A. marajoara*. Haplotypes 62-67 (light grey) are *A. oryzalimnetes *from GenBank only; and S represents the corresponding collection sites (see Table 1).

The estimated MP and BI trees, each with 42 parsimoniously informative sites, indicated two distinct lineages of *A. marajoara *with varying levels of support (Figure 1). The more derived lineage 1 remains ambiguous (only moderate support, 0.66 and 52%); however it does have the broadest distribution and contains samples from near (~56 km) the type locality on Marajó Island, Pará state, Brazil [79]. Lineage 2, in contrast, is more restricted geographically and has higher support (1.00 and 89%, Figure 1). The relatively low BI branch support (0.66) for *A. marajoara *lineage 1 coupled with the moderate support among some of the subdivisions suggests this lineage is paraphyletic and that haplotypes that would otherwise increase lineage support are missing (extinct or not sampled). Within both lineages, smaller subdivisions, not related to geography, were apparent between the haplotypes that are separated by seven mutations in lineage 1 (between haplotypes 11 and 13) and 10 mutations in lineage 2 (haplotypes 48 and 49), illustrated in the network (Figure 2).

The estimated time to coalescence for the two *COI *lineages was obtained using the equations, *θ*_*s *_= 2*Neμ*, [29] and 4*N*_*e *_*= *years since coalescence [80]. Based upon the 488-bp fragment, *θ*_*s *_is 7.530 (SD = 1.857). Therefore, the estimate of the time to coalescence is 232,476 - 384,716 years ago, in the Pleistocene. A similar estimate using BEAST, the standard arthropod mtDNA mutation rate of 2.3% per million years [81], an applied HKY model with gamma distribution assuming constant size and a relaxed molecular clock, yielded an estimate of 3.07 mya. The discrepancy between coalescent estimates is likely due to the 10 generations per year that is factored into the Walton equation [29]. Although the incorporation of fossil calibration points can generate more reliable divergence estimates [82], no data were available because the mosquito fossil record is too recent and rare [83].

### Genetic variation

The between lineage variance for the Folmer region of the two lineages of *A. marajoara *(2.94%) is marginally below the 3% BOLD threshold. However, it is comparable to among species comparisons within the Albitarsis Complex (e.g., *A. albitarsis *s.s. vs. *A. oryzalimnetes*, 2.64%; Additional file 2). The 3' end of the *COI *(above diagonal, Additional file 2) is less conserved, resulting in higher estimates of divergence. Among closely related *Nyssorhynchus *spp. (Table 2), the average intraspecific variation is 0.0116, and suggests that a SST of 11.6% might suffice within this subgenus. However, this estimate is limited as only 7 out of 32 currently recognized species [65] were available. Intraspecific nucleotide differences among *Nyssorhynchus *species were consistently less than 2%, indicating that the higher estimate (0.021) of combined *A. marajoara *lineages likely represents an overlooked species complex.

**Table 2:** *COI *K2P intraspecific nucleotide difference of *Nyssorhynchus *species using the fragment of the Folmer region available in GenBank with current *A. marajoara *and individual lineage comparisons below the double line.

Species	N	Fragment length	Intraspecific difference mean (SD)	GenBank Accession
*A. albitarsis s.s*	6	703	0.010 (± 0.003)	DQ076204-DQ076209

*A. oryzalimnetes*	6	703	0.004 (± 0.002)	DQ076210-DQ076215

*A. deaneorum*	4	703	0.015 (± 0.003)	DQ076226/227, DQ076229/230

*A. goeldii*	16	493	0.014 (± 0.003)	EU848313-EU848328

*A. braziliensis*	58	529	0.015 (± 0.003)	DQ913858-DQ913877

*A. darlingi*	36	460	0.012 (± 0.003)	DQ298209-DQ298244

*A. dunhami*	4	493	0.010 (± 0.003)	EU848329-EU848332

*A. marajoara s.l*	10	703	0.024 (± 0.003)	DQ076216-DQ076225

Lineage 1	7	703	0.016 (± 0.002)	DQ07616, DQ076218-20, DQ076222-24

Lineage 2	3	703	0.014 (± 0.002)	DQ076217, DQ076221, DQ076225

**Figure 3:**
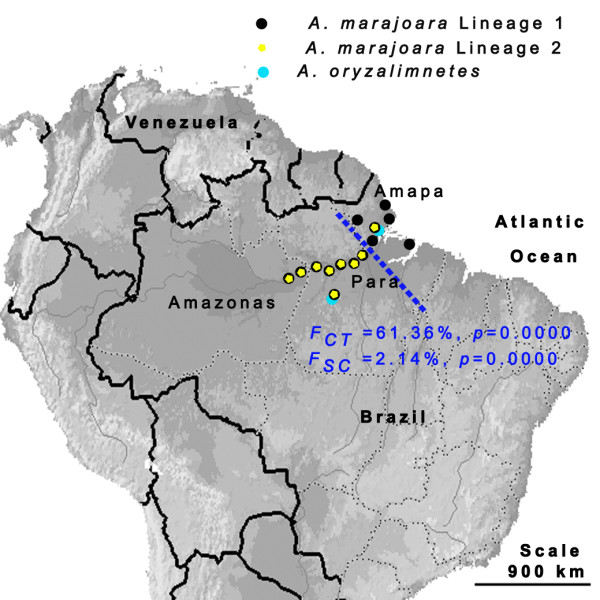
**Distribution of collection localities of *A. marajoara *s.l. and *A. oryzalimentes***. Map of South America indicating the location of the 14 collection localities, lineage distributions and the spatial grouping derived from the SAMOVA analysis. Dotted line corresponds to SAMOVA defined groupings (eastern and western Amazon), based on sequence similarity and geographic distance for *A. marajoara *lineage 1 among all localities. *F*_*CT *_corresponds to between group variation and *F*_*SC *_indicates variation within groupings.

AMOVA indicated that 61.94% (*p *= 0.0000) of the variance was explained by between-group variation of *A. marajoara *lineages 1 and 2. SAMOVA analysis, providing resolution for lineage 1 only, defined two groups that correspond to northeastern and western Amazonia, with 61.36% regional variation (Figure 3). Several *COI *haplotypes were found in both the northeastern and western populations of lineage 1 indicating at least some gene flow across the geographic barrier. High variation, especially between lineages 1 and 2, and between geographic populations in both *COI *and *white *genes, is strongly supported by population differentiation statistics, although the *G*_*ST *_values were not significantly different (Table 3). Furthermore, the *K*_*t *_values were much greater for the *COI *gene (11.9 and 6.96), compared with the *white *gene (1.0).

**Table 3:** Inter- and intra-population differentiation of *A. marajoara *lineages and populations on either side of the SAMOVA generated boundary.

	*COI *(488-bp fragment)		*white *(496 bp)
	
	Lineage 1 vs. 2 (*N *= 289)	Lineage 1 E vs. W (*N *= 209)	East vs. West
**H**_**s**_	0.81757***	0.71449***	0.65635***

**K **_s _*	1.66659***	1.05701***	0.99335***

Z*	9.19220***	8.65007***	7.70914***

**S**_**nn**_	0.99301***	0.8298***	0.82814***

**χ**^**2**^	282.612***	148.927***	59.991***

***G*st**	0.08536	0.13799	0.05827

***k***_**t**_	11.92573	6.95565	1.03981

*N*, number of total individuals compared; *H*_*S*_, genetic differentiation based on haplotype data; *K*_S_*, differentiation based on sequence data; Z*, rank statistic to analyze sequence similarity; *S*_*nn*_, measures how often the nearest neighbours of sequences are found in the same population; *c*^*2*^, genetic differentiation based on allele frequencies; *G*_*ST*_, genetic differentiation; *K*_*t*_, average number of nucleotide differences; ** significance, *p *< 0.02; *** significance, *p *< 0.0001

### Population structure and demographic history

Analysis of mitochondrial data from 294 samples among seven riverine and seven non-riverine localities in Amazonas, Pará, and Amapá states, Brazil (Figure 3) led to the discovery of two co-occuring, but distinct *A. marajoara *lineages that are unable to be connected using statistical parsimony (Figure 2). Therefore, a median joining network that uses alternate algorithms to remove homoplasy was conducted and indicated that a minimum of 13 mutational steps separate the lineages, shown in Figure 2. Seven fixed mutations were estimated between lineages based on DnaSP. Additionally, *A. oryzalimenetes *was identified from Itaituba and from Macapá, a new record (Figure 3).

*COI *haplotypes were detected (Figure 2). Of the *A. marajoara *haplotypes, 26 (44.8%) were shared among localities, and 32 (55.2%) were unique (Additional file 3). The most common haplotypes were 1 (*n *= 16), 2 (*n *= 26), 3 (*n *= 20) and 5 (*n *= 78) of *A. marajoara *lineage 1, and haplotypes 43 (*n *= 9) and 46 (*n *= 9), 50 (*n *= 32), and 56 (*n *= 11) of *A. marajoara *lineage 2 (Figure 2). There was a relatively high proportion of singletons (32, 55.17%) in the *A. marajoara *lineages combined, with 24/42 for lineage 1, and 9/16 for lineage 2. Overall, both *A. marajoara *lineages were indicative of a category II pattern [26] characterized by pronounced genetic gaps between some branches and the co-distribution of principle lineages over a wide area, which could theoretically arise in a species with large evolutionary *N*_*e *_(effective population size) and high gene flow. Lineage 1 contains a star shaped node surrounding haplotype 5, with short branches and an excess of singleton mutations, predominantly from the northeastern localities, which suggests a demographic expansion, background selection or selective sweep [74,84]. In contrast, lineage 2 indicates balancing selection with its longer branches, missing haplotypes and near equal distribution of shared haplotypes and single mutations, consistent with a signal of an older lineage. It could be argued that haplotype 50 (lineage 2, Figure 2) and the 5 singletons that arise from it also constitute a star-shaped node, which suggest recent expansion. Lineage 2 appears to be restricted to the westernmost localities.

**Figure 4:**
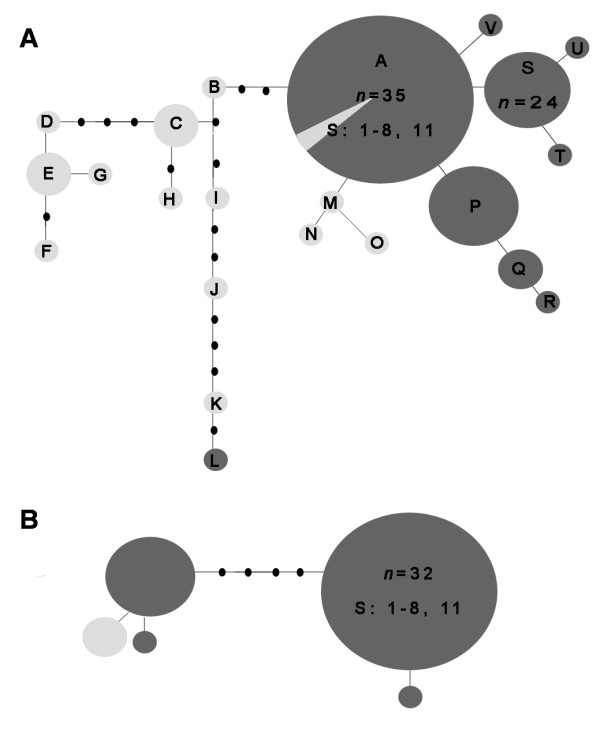
**Parsimony-based haplotype networks of nuclear *white *and ITS2 for *A. marajoara***. Statistical parsimony network (95%): A) 107 *white *gene sequences and their corresponding haplotypes and B) 53 ITS2 sequences. There was no distinction between lineages; grey haplotypes are all from western localities (1-8 Figure 1; Table 1) and light-colored haplotypes are all from northeastern localities (9-14).

Statistical parsimony networks of nuclear data (*white *gene and ITS2) containing comparative numbers of specimens of both *COI*-defined lineages identified a single *A. marajoara *lineage (Figure 4A, B). Haplotype clusters (Figure 4A) correspond primarily to northeastern and western populations with a few outliers including haplotype L and two individuals in haplotype A. Both lineages retained the 4^th ^intron in the *white *gene, which is absent from all other Albitarsis Complex members [11]. Additionally, the ITS2 length was identical (361-bp) in all sequences, independent of the lineage. Copy number in microsatellite regions at positions 118 (GT) and 273 (GA) and the 2-bp indel at position 271 were not congruent with either lineage or geography, but consistent with findings by Li and Wilkerson [20,42]. Genetic polymorphism analyses of the *COI *sequences indicated that haplotype diversity was greater among lineage 1 populations, whereas lineage 2 localities exhibited slightly higher nucleotide diversity (Additional file 3). In lineage 1, there was greater diversity among the western localities (1-8), compared to northeastern Amazon (9-14). The *white *gene exhibited lower nucleotide diversities overall compared to *COI*, and some differences in diversity between populations, particularly in Tartarugalzinho (locality 12), where haplotypes D and T are present, separated by 11 mutation steps (Additional file 4). Moderate nucleotide diversity in the *COI *data coupled with the single *A. marajoara *lineage detected with the *white *gene may reflect ancestral polymorphism as a result of co-occurence and haplotype mixing [85].

The high *COI *haplotype diversity combined with relatively low nucleotide diversity in both lineages (Additional file 3) suggests a population bottleneck followed by an expansion. The negative values for three neutrality tests in lineage 1 (Table 4) may reflect an excess of rare polymorphisms consistent with either positive selection or an increase in population size, although only one of these tests (*F*_*s*_) was significant. In contrast, the positive values in lineage 2 indicate an excess of intermediate-frequency alleles in a population, which can result from either balancing selection or population bottlenecks [86]. Only the northeastern subdivision of lineage 1 revealed strong support for a population expansion event. The comparison between the northeastern and western populations within lineage 1 supports the SAMOVA findings of a geographic barrier, depicted in Figure 3.

**Table 4:** Results of neutrality tests based on *COI *sequences of *A. marajoara *from Amazonian Brazil.

*A. marajoara*	*N*	***D***_***T***_	D*	F*	***F***_***S***_	***R***_***2***_
Lineage 1	209	0.92686	-1.44039	-0.54355	-9.637*	0.1147

Western	122	0.58594	-0.61517	-0.17709	-6.611*	0.1101

Eastern	87	-2.40421**	-1.70059	-2.5166*	-15.498**	0.0214**

Lineage 2	80	2.52378*	0.52692	1.50581	1.920	0.1843

*N*, sample size; D_T_, Tajima's D; D, Fu and Li's D; F, Fu and Li's F; F_S_, Fu's Fs; R2, Ramos-Onsin's and Rosa's; * significance, *p *< 0.50; ** significance, *p *< 0.02.

**Figure 5:**
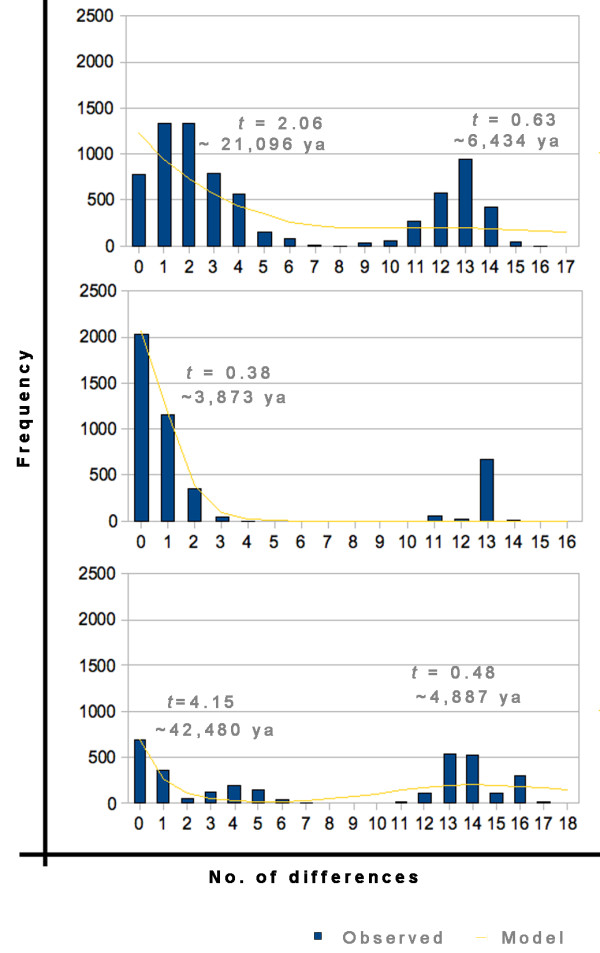
**Mismatch distributions of pairwise sequence differences in *A. marajoara *among lineages and geographic populations**. Representative mismatch distributions for *A. marajoara *lineages with calculated *tau *and estimated time of spatial expansion. Black bars indicate observed values, the grey line represents the model. A) *A. marajoara *lineage 1 across all localities; B) *A. marajoara *lineage 1 from the 6 northeastern localities; and C) *A. marajoara *lineage 2.

The mismatch distribution model for sudden expansion was marginally significant for both lineages. Both exhibited a bi-modal distribution (Figure 5) with a complete separation and significant raggedness values, suggesting constant population size [87]. However, an alternative interpretation would be that there were two expansions [88] dated to the Pleistocene (lineages 1 and 2, 142,203 (3,678-220,163) and 114,713 (8,545-230,635) ybp, respectively). The lineage 1 northeastern population (Figure 2) expanded more recently, approximately 5,000 years ago, during the Holocene.

## Discussion

The combined sequence results support divergence in *A. marajoara *depicting separate lineages, distinct from *A. janconnae*. The more ancestral lineage 2 appears monophyletic, whereas moderate substructure within lineage 1 suggests paraphyly. Nuclear markers, however, consistently depict a single *A. marajoara *lineage. Discrepancies between mtDNA and nDNA are not enough to refute speciation, for example the newly recognized species *A. janconnae *and *A. marajoara *form a single lineage using the protein coding nuclear *white *gene and ITS2 sequences [20,21], but complete *COI *sequences [10] indicate monophyly of each taxon, and morphometrics analysis depicts *A. janconnae *as a separate taxon in the complex [22]. Nuclear mutation rates are generally slower than mitochondrial ones [89], but see Hellberg [90], thus the lack of divergence detected in the nuclear sequences in *A. marajoara *in the present study suggests either the differentiation is restricted to the mitochondrial genome, or it is recent and not yet visible in the nuclear genome. Given that lineage 1 has a broader distribution and includes samples from near the type locality, lineage 1 would be more appropriately named *A. marajoara *s.s. The status of lineage 2 is not yet resolved; it may be a new cryptic species belonging to the complex as suggested by Wilkerson *et al*. (2005) from Manaus, Brazil [41] and confirmed by JF Ruiz (personal communication) using the *COI *barcode region of the Albitarsis subgroup across South America.

Genetic variation between lineages was explained by 61.94% between group differences. Pairwise divergence estimates based on the 488-bp fragment confirmed 3.82% divergence between lineages (Table 3), which is comparable to the values observed among well-known Albitarsis Complex species (for example, 3.52% between *A. albitarsis s.s *and *A. oryzalimnetes*). These data may support cryptic or incipient speciation [91]. The effectiveness of the Folmer region and the BOLD threshold of 3% are questionable for at least some species of *Nyssorhynchus *because there is only moderate support between known species *A. oryzalminetes *and *A. albitarsis *s.s. If the average intraspecific variation of the Folmer region for the subgenus (11.6%) is used as a species threshold, the pairwise divergences of all sister taxa (including *A. darlingi *and *A. albimanus*) do not exceed this standard. Such a high SST is likely to result in false negatives even among recognized species, therefore separate intraspecific values and interspecific divergences were examined for resolution, where intraspecific *COI *variation was <2% and interspecific variation was between 6-10%, comparable to other anopheline species differences [[92,93], unpublished data].

Pleistocene divergence in mtDNA has also been detected for a wide range of insect taxa across Central and South America, including *L. longipalpis *[94], *H. erato *[81], *R. prolixus *[95], and mosquitoes *A. darlingi *[53] and *A. albimanus *[85] suggesting climatic changes may be a common force driving Neotropical speciation. Pleistocene divergence coupled with paleoclimate records of the Miocene describing the world as warmer and exhibiting greater humidity and precipitation [96]; [97] support the more ancestral depiction of lineage 2. Pleistocene climatic changes, including lower temperature, precipitation and carbon dioxide levels than those exhibited today, may have contributed to the divergence of lineage 1 [98,99].

Lineages co-occur along the Amazon River with no obvious species diversity gradients, although data indicate several "high diversity localities" including Santarém, which is a recognized hotspot. However, this may be an artefact resulting from the relative logistical ease of access to these localities and the many studies that have been conducted there [100]. The geographic barrier detected within *A. marajoara *lineage 1 is in the same region as the mtDNA divisions between northeastern and central western Amazon populations in *A. darlingi *[101], *A. nuneztovari *[102] and *Atta *species ants [103]. With similar distributions and geographic boundaries noted in both mosquitoes and ants, it seems unlikely that the boundary is the result of dispersal abilities. The detection of shared haplotypes in the present study on both sides of the barrier indicates a permeable boundary, suggesting the more geographically restricted lineage 2 may be an artefact of incomplete sampling, or perhaps a result of climate variation.

Climate, particularly rainfall, is a strong descriptor of broad-scale species-richness patterns in the tropics [104]. Vasconcelos *et al *[105] noted a restricted geographic distribution of ants in the western part of the basin, which to a great extent reflected a west to east gradient of decreasing rainfall. Additionally, Sombroek [106] explained an intricate pattern of continuous rainfall in the western interior of the Amazon, in contrast to the coasts, with a pronounced dry season containing a dry belt or corridor occurring near Rio Jari, Amapá state (near the SAMOVA barrier for *A. marajoara *lineage 1). Miocene flooding [103] and subsequent Pleistocene climatic events may explain the porous nature of the barrier.

Recent deforestation and habitat fragmentation resulting from the 1967 initiation of the single largest forest plantation in Latin America, an agro-forestry venture known as the Jari project [107] in northern Brazil could have contributed to an increase in *A. marajoara *abundance in this region. Clearing of forest, in combination with an increase in human and domestic animal host abundance, may have led to an *A. marajoara *population increase in Macapá, the capital of Amapá -state, in northeastern Amazonian Brazil [15].

Among the riverine localities, the hypothesis of sudden population expansion could not be rejected, although the mismatch distributions were smooth and bimodal for both lineages. The reduced genetic variation of *A. marajoara *lineage 1 in the northeastern Amazon region coupled with the unimodal distribution and the star-like pattern of the haplotype network are consistent with a rapid population expansion in the northeast. *A. marajoara *tends to breed in marshy sunlit pools and may have expanded into the northeast where savannah and agricultural habitats would be favorable. The population expansion occurred during the Holocene, possibly indicating a recent colonization. This explanation is supported by the expansion of savannah during periods of fire-associated drought and extinction-recolonization of rainforest tree populations in lowland areas during the Holocene [108-110]. Moderate gene flow in lineage 1 may hamper local vector control in Amazonas, Pará and Amapá states because of potential reinvasion and the spread of insecticide resistant genes [111].

## Conclusions

*Anopheles marajoara *is an important vector in the savannah areas of South America [13], and with some of the highest incidences of malaria occurring in the states of Pará and Amapá [14,15,112] it is likely that *A. marajoara *will continue to increase its regional importance in malaria transmission. Additional studies will be needed to determine whether the subdivision resulting in the two co-occurring lineages in *A. marajoara *is strictly the product of genetic variation and evolutionary processes impacted by geographical barriers, or variations in ecology and behaviour that may have lead to niche divergences and microallopatry [113-116]. If both lineages are implicated as vectors, the characteristics of their breeding sites, their behaviours and their migration history could be used to predict changes in malaria transmission patterns in the Amazon basin [15] and other endemic regions, and provide useful information for targeted vector control strategies.

## Competing interests

The authors declare that they have no competing interests.

## Authors' contributions

SNM extracted DNA, performed all molecular procedures and analysis, and drafted the manuscript. MAL performed molecular procedures and analysis for eastern data and helped in manuscript preparation. RCW and MMP contributed in the design of the study, were involved in field collections and helped interpret data. JFR assisted in data analysis and interpretation. MAS and JBLP were involved in field collections and performed morphological identifications of samples with RCW. JEC participated in the design of the study, data analysis, drafting of the manuscript, general supervision of the research group and funding acquisition. All authors read and approved the final document.

## Supplementary Material

Additional file 1 **Complete *COI *with fragment orientation and overlap**. Representative schematic of the *COI *gene, Folmer region and fragments; light blue bar indicating the region amplified by primers UEA3 and UEA10; the purple bar depicting fragment previously amplified from primers 2195D and C1-J-2195. *, denotes the piece of the *COI *that was used for the phylogeography and population structure analysis. Click here for file

Additional file 2 ***D*_*XY *_with Jukes Cantor pairwise divergence between species and lineages based upon the 648-bp Folmer region (below) and the 488-bp COI fragment (above)**. Click here for file

Additional file 3 **Description of shared *COI *haplotypes and genetic polymorphism statistics for *A. marajoara *lineages and *A. oryzalimnetes***. *N*, the number of sequences; *1-Σf*_*i*_^*2*^, haplotype diversity; *Π*, nucleotide diversity; SD, standard deviation; P, polymorphic sites; and *K*, average number of differences. Underlining indicates shared haplotypes and bold indicates diversity among lineages or species. Click here for file

Additional file 4 **Description of shared *white *haplotypes and genetic polymorphism statistics for *A. marajoara *s.l**. Click here for file

## References

[B1] Hammond PM Groombridge B Species inventory Global biodiversity: status of the earth's living resources 1992 Chapman & Hall, London 17 39

[B2] Lewinsohn TM Prado PI Biodiversity of Brazil: a synthesis of the current state of knowledge in Biodiversidade brasileira: síntese do estado do conhecimento atual 2002 Contexto Acadêmica, São Paulo 139 144

[B3] De Quieroz K Species concepts and species delimitation Syst Biol 2007 56 879 886 10.1080/10635150701701083 18027281

[B4] Bickford D Lohman DJ Sodhi NS Ng PKL Meier R Winker K Ingram KK Das I Cryptic species as a window on diversity and conservation Trends Ecol Evol 2006 22 148 155 10.1016/j.tree.2006.11.004 17129636

[B5] Gomez A Serra M Carvalho GR Lunt DH Speciation in ancient cryptic species complexes: Evidence from the molecular phylogeny of *Brachionus plicatilis *(Rotifera) Evol 2002 56 1431 1444 10.1111/j.0014-3820.2002.tb01455.x 12206243

[B6] Olsson U Alstrom P Ericson PGP Sundberg P Non-monophyletic taxa and cryptic species - evidence from a molecular phylogeny of leaf-warblers (*Phylloscopus*, Aves) Mol Phylogenet Evol 2005 36 261 276 10.1016/j.ympev.2005.01.012 15955509

[B7] Illera JC Richardson DS Helm B Atienza JC Emerson BC Phylogenetic relationships, biogeography and speciation in the avian genus *Saxicola* Mol Phylogenet Evol 2008 48 1145 1154 10.1016/j.ympev.2008.05.016 18571939

[B8] Pfenninger M Nowak C Kley C Steike D Streit B Utility of DNA taxonomy and barcoding for the inference of larval community structure in morphologically cryptic *Chironomus *(Diptera) species Mol Ecol 2007 16 1957 1968 10.1111/j.1365-294X.2006.03136.x 17444904

[B9] Lins RMMA Souza NA Peixoto AA Genetic divergence between two sympatric species of the *Lutzymyia longipalpis *complex in the paralytic gene, a locus associated with insecticide resistance and lovesong production Mem Inst Oswaldo Cruz 2008 103 736 740 10.1590/S0074-02762008000700019 19057828

[B10] Lehr MA Kilpatrick CW Wilkerson RC Conn JE Cryptic species in the *Anopheles *(*Nyssorhynchus*) *albitarsis *(Diptera: Culcidae) complex: incongruence between random amplified polymorphic DNA-polymerase chain reaction identification and analysis of mitochondrial DNA *COI *gene sequences Ann Entomol Soc Am 2005 98 908 917 10.1603/0013-8746(2005)098[0908:CSITAN]2.0.CO;2 17082822 PMC1633725

[B11] Merritt TJ Young CR Vogt RG Wilkerson RC Quattro JM Intron retention identifies a malaria vector within the *Anopheles *(*Nyssorhynchus*) *albitarsis *complex (Diptera: Culicidae) Mol Phylogenet Evol 2005 35 719 724 10.1016/j.ympev.2005.03.009 15878139

[B12] Dusfour I Blondeau J Harbach RE Vythilingham I Baimai V Trung HD Sochanta T Bangs MJ Manguin S Polymerase chain reaction identification of three members of the *Anopheles sundaicus *(Diptera: Culicidae) complex, malaria vectors in Southeast Asia J Med Entomol 2007 44 723 31 10.1603/0022-2585(2007)44[723:PCRIOT]2.0.CO;2 17915501

[B13] Rubio-Palis Y Zimmerman RH Ecoregional classification of malaria vectors in the Neotropics J Med Entomol 1997 34 499 510 9379453 10.1093/jmedent/34.5.499

[B14] Povoa MM Wirtz RA Lacerda RNL Miles MA Warhurst D Malaria vectors in the municipality of Serra do Navio, state of Amapá, Amazon region, Brazil Mem Inst Oswaldo Cruz 2001 96 179 184 10.1590/S0074-02762001000200008 11285494

[B15] Conn JE Wilkerson RC Segura MNO De Souza RTL Schlichting CD Wirtz RA Povoa MM Emergence of a new neotropical malaria vector facilitated by human migration and changes in land use Am J Trop Med Hyg 2002 66 18 22 12135261 10.4269/ajtmh.2002.66.18

[B16] Povoa MM De Souza RTL Nonato da Luz Lacerda R Santa Rosa E Galiza D Rodrigues de Souza J Wirtz R Schlichting C Conn JE The importance of *Anopheles albitarsis E *and *An. darlingi *in human malaria transmission in Boa Vista, state of Roraima, Brazil Mem Inst Oswaldo Cruz 2006 101 163 168 10.1590/S0074-02762006000200008 16830709

[B17] Bourke BP Foster PG Bergo ES Calado DC Sallum MAM Phylogenetic relationships among species of *Anopheles *(*Nyssorhynchus*) (Diptera: Culicidae) based on nuclear and mitochondrial gene sequences Acta Trop 2010 114 88 96 10.1016/j.actatropica.2010.01.009 20117069

[B18] Klein TA Lima JB Tada MS Comparative susceptibility of anopheline mosquitoes to *Plasmodium falciparum *in Rondonia, Brazil Am J Trop Med Hyg 1991 44 598 603 1858963 10.4269/ajtmh.1991.44.598

[B19] Klein TA Lima JB Tada MS Miller R Comparative susceptibility of anopheline mosquitoes in Rondonia, Brazil to infection by *Plasmodium vivax* Am J Trop Med Hyg 1991 45 463 470 1951854 10.4269/ajtmh.1991.45.463

[B20] Li C Wilkerson RC Identification of A*nopheles (Nyssorhynchus) albitarsis *complex species (Diptera:Culicidae) using rDNA internal transcribed spacer 2-based polymerase chain reaction primers Mem Inst Oswaldo Cruz 2005 100 495 500 10.1590/S0074-02762005000500009 16184227

[B21] Brochero HL Li C Wilkerson RC A newly recognized species in the *Anopheles *(*Nyssorhynchus*) *albitarsis *complex (Diptera: Culcidae) from Puerto Carreno, Colombia Am J Trop Med Hyg 2007 76 1113 1117 17556620

[B22] Motoki MT Wilkerson RC Sallum MAM The *Anopheles albitarsis *complex with the recognition of *Anopheles oryzalimnetes *Wilkerson and Motoki, n. sp. and *Anopheles janconnae *Wilkerson and Sallum, n. sp. (Diptera: Culicidae) Mem Inst Oswaldo Cruz 2009 104 823 850 10.1590/S0074-02762009000600004 19876554

[B23] Wiens JJ Species delimitation: New approaches for discovering diversity Syst Biol 2007 56 875 878 10.1080/10635150701748506 18027280

[B24] Bowling AT Ruvinsky A The Genetics of the Horse 2000 CABI Publishing, Wallingford

[B25] Avise JC Molecular markers, natural history and evolution 1994 Chapman & Hall, New York

[B26] Avise JC Phylogeography. The History and Formation of Species 2000 University Press: Massachusetts

[B27] Brown JM Pellmyr O Thompson JN Harrison RG Phylogeny of *Greya *(Lepidoptera: Prodoxidae), based on nucleotide sequence variation in the mitochondrial cytochrome oxidase I and II: congruence with morphological data Mol Biol Evol 1994 11 128 141 8121281 10.1093/oxfordjournals.molbev.a040087

[B28] Lunt DH Zhang DX Szymura JM Hewitt GM The insect cytochrome oxidase I gene: evolutionary patterns and conserved primers for phylogenetic studies Insect Mol Biol 1996 5 153 165 10.1111/j.1365-2583.1996.tb00049.x 8799733

[B29] Walton C Handley JM Tun-Lin W Collins FH Harbach RE Baimai V Butlin RK Population structure and population history of *Anopheles dirus *mosquitoes in Southeast Asia Mol Biol Evol 2000 17 962 974 10833203 10.1093/oxfordjournals.molbev.a026377

[B30] Salvato P Battisti A Concato S Masutti L Patarnello T Zane L Genetic differentiation in the winter pine processionary moth (*Thaumetopoea pityocampa-wilkinsoni *complex) inferred by AFLP and mitochondrial DNA markers Mol Ecol 2002 11 2435 2444 10.1046/j.1365-294X.2002.01631.x 12406253

[B31] Hebert PDN Penton EH Burns JM Janzen DH Hallwachs W Ten species in one: DNA barcoding reveals cryptic species in the neotropical skipper butterfly *Astraptes fulgerator* Proc Natl Acad Sci USA 2004 101 14812 14817 10.1073/pnas.0406166101 15465915 PMC522015

[B32] Cooper JK Sykes G King S Cottrill K Ivanova NV Hanner R Ikonomi P Species identification in cell culture: a two-pronged molecular approach In Vitro Cell Dev Biol Anim 2007 43 344 351 10.1007/s11626-007-9060-2 17934781

[B33] Hebert PDN Cywinska A Ball SL deWaard JR Biological identifications through DNA barcodes Proc R Soc Lond B Biol Sci 2003 270 313 321 10.1098/rspb.2002.2218 PMC1691236 12614582

[B34] Moritz C Cicero C DNA Barcoding: Promise and pitfalls PLoS Biol 2004 2 1529 1531 10.1371/journal.pbio.0020354 PMC519004 15486587

[B35] Ball SL Hebert PDN Burian SK Webb JM Biological identifications of mayflies (Ephemeroptera) using DNA barcodes J N Am Benthol Soc 2005 24 508 524

[B36] Foley DH Wilkerson RC Cooper RD Volovsek ME Bryan JH A molecular phylogency of *Anopheles annulipes *(Diptera: Culicidae) sensu lato: The most species-rich anopheline complex Mol Phylogenet Evol 2006 43 283 297 10.1016/j.ympev.2006.10.008 17126567

[B37] Whitworth TL Dawson RD Magalon H Baudry E DNA barcoding cannot reliably identify species of the blowfly genus *Protocalliphora *(Diptera: Calliphoridae) Proc R Soc Biol Sci 2007 274 1731 1739 10.1098/rspb.2007.0062 PMC2493573 17472911

[B38] Elias M Hill RI Willmott KR Dasmahapatra KK Brower AVZ Mallet J Jiggins CD Limited performance of DNA barcoding in a diverse community of tropical butterflies Proc R Soc Biol 2007 274 2881 2889 10.1098/rspb.2007.1035 PMC3227132 17785265

[B39] Meyer CP Paulay G DNA Barcoding: Error rates based on comprehensive sampling PLoS Biol 2005 3 e422 10.1371/journal.pbio.0030422 16336051 PMC1287506

[B40] Cognato AI Standard percent DNA sequence difference for insects does not predict species boundaries J Econ Entomol 2006 99 1037 1045 10.1603/0022-0493-99.4.1037 16937653

[B41] Wilkerson RC Foster PG Li C Sallum MAM Molecular phylogeny of the neotropical *Anopheles (Nyssorhynchus) albitarsis *species complex (Diptera: Culicidae) Ann Entomol Soc Am 2005 98 918 925 10.1603/0013-8746(2005)098[0918:MPONAN]2.0.CO;2 18079976 PMC2134802

[B42] Li C Wilkerson RC Intragenomic rDNA ITS2 variation in the neotropical *Anopheles (Nyssorhynchus) albitarsis *Complex (Diptera: Culicidae) J Hered 2007 98 51 59 10.1093/jhered/esl037 17158469

[B43] Fritz GN Conn JE Cockburn AF Seawright J Sequence analysis of the ribosomal DNA Internal Transcribed Spacer 2 from populations of *Anopheles nuneztovari *(Diptera: Culicidae) Mol Biol Evol 1994 11 406 416 8015435 10.1093/oxfordjournals.molbev.a040122

[B44] Marrelli MT Sallum MAM Marinotti O The second internal transcribed spacer of the nuclear ribosomal DNA as a tool for Latin American anopheline taxonomy - a critical review Mem Inst Oswaldo Cruz 2006 101 817 832 10.1590/S0074-02762006000800002 17293975

[B45] Zapata MA Cienfuegos AV Quiros OI Quinones ML Luckhard S Correa MM Discrimination of seven *Anopheles *species from San Pedro de Uraba, Antioquia, Colombia, by Polymerase Chain Reaction-Restriction Fragment Length Polymorphism analysis of ITS sequences Am J Trop Med Hyg 2007 77 67 72 17620632

[B46] Marrelli MT Floeter-Winter LM Malafronte RS Tadei WP Lourenc R O-De-Oliverira Flores-Mendoza C Marinotti O Amazonian malaria vector anopheline relationships intrepreted from ITS2 rDNA sequences Med Vet Entomol 2005 19 208 218 10.1111/j.0269-283X.2005.00558.x 15958027

[B47] Hartl DL Clark AG Principles of Population Genetics 2007 4 Sinauer Associates, Inc. Publishers, Sunderland MA

[B48] Caporaso JG Smit S Easton BC Hunter L Huttley GA Knight R Detecting coevolution without phylogenetic trees? Tree-ignorant metrics of coevolution perform as well as tree-aware metrics BMC Evol Biol 2008 8 327 10.1186/1471-2148-8-327 19055758 PMC2637866

[B49] Deane LM Causey OR Deane MP An illustrated key by adult female characteristics for identification of thirty-five species of *Anophelini *from the Northeast and Amazon regions of Brazil, with notes on the malaria vectors (Diptera: Culicidae) Am J Hyg Monog Ser 1946 18 1 18

[B50] Lehr MA Inter- and intra-specific relationships of the cryptic members of the *Anopheles *(*Nyssorhynchus*) *albitarsis *species complex Masters thesis 2003 University of Vermont, Biology Department

[B51] Soto-Adames FN Molecular phylogeny of the Puerto Rican *Lepidocyrtus *and *pseudosinella *(Hexapoda: Collembola: Entomobryidae), a validation of Yoshii's "color pattern species" Mol Phylogenet Evol 2002 25 27 42 10.1016/S1055-7903(02)00250-6 12383748

[B52] Joy DA Conn JE Molecular and morphological phylogenetic analysis of an insular radiation in Pacific black flies (*Simulium*) Syst Biol 2001 50 18 38 10.1080/106351501750107431 12116592

[B53] Mirabello L Conn JE Molecular population genetics of the malaria vector *Anopheles darlingi *in Central and South America Hered 2006 96 311 321 10.1038/sj.hdy.6800805 16508661

[B54] Swofford DL PAUP (Phylogenetic Analysis Using Parsimony) and other methods, Version 4 2004 Sinauer Associates: Massachusetts

[B55] Mirabello L Conn JE Population analysis using the nuclear *white *gene detects Pliocene/Pleistocene lineage divergence within *Anopheles nuneztovari *in South America Med Vet Entomol 2008 22 109 19 10.1111/j.1365-2915.2008.00731.x 18498609

[B56] Huelsenbeck JP Ronquist F MRBAYES: Bayesian inference of phylogeny Bioinformat 2001 17 754 755 10.1093/bioinformatics/17.8.754 11524383

[B57] Ronquist F Huelsenbeck JP MRBAYES 3: Bayesian phylogenetic inference under mixed models Bioinformat 2003 19 1572 1574 10.1093/bioinformatics/btg180 12912839

[B58] Posada D jModelTest: Phylogenetic model averaging Mol Biol Evol 2008 25 1253 1256 10.1093/molbev/msn083 18397919

[B59] Guindon S Gascuel O A simple, fast, and accurate algorithm to estimate large phylogenies by maximum likelihood Syst Biol 2003 52 696 704 10.1080/10635150390235520 14530136

[B60] Sallum MAM Schultz TR Wilkerson RC Phylogeny of anophelinae (Diptera Culicidae) based on morphological characters Ann Entomol Soc Am 2000 93 745 775 10.1603/0013-8746(2000)093[0745:POADCB]2.0.CO;2

[B61] Watterson GA On the number of segregating sites in genetic models without recombination Theor Popul Biol 1975 7 256 276 10.1016/0040-5809(75)90020-9 1145509

[B62] Drummond AJ Rambaut A "BEAST: Bayesian Evolutionary Analysis by Sampling Trees." BMC Evol Biol 2007 7 214 10.1186/1471-2148-7-214 17996036 PMC2247476

[B63] Excoffier L Laval G Schmeider S Arlequin ver 3.0: An integrated software package for population genetic data analysis Evol Bioinformat Online 2005 1 47 50 PMC2658868 19325852

[B64] Dupanloup I Schneider S Excoffier L A simulated annealing approach to define the genetic structure of populations Mol Ecol 2002 11 2571 81 10.1046/j.1365-294X.2002.01650.x 12453240

[B65] Harbach RE The classification of genus *Anopheles *(Diptera: Culicidae): a working hypothesis of phylogenetic relationships Bull Entomol Res 2004 95 537 553 10.1079/ber2004321 15541193

[B66] Witt JDS Threloff DL Hebert PDN DNA barcoding reveals extraordinary cryptic diversity in an amphipod genus: implications for desert spring conservation Mol Ecol 2006 15 3073 3082 10.1111/j.1365-294X.2006.02999.x 16911222

[B67] Hebert PDN Stoeckle MY Zemlak TS Francis CM Identification of birds through DNA barcodes PloS Biol 2004 2 e312 10.1371/journal.pbio.0020312 15455034 PMC518999

[B68] Clement M Posada D Crandall KA TCS: a computer program to estimate gene genealogies Mol Ecol 2000 9 1657 1659 10.1046/j.1365-294x.2000.01020.x 11050560

[B69] Crandall KA Templeton AR Empirical tests of some preditions from coaslescent theory with applications to intraspecific phylogeny reconstruction Genet 1993 134 959 969 10.1093/genetics/134.3.959 PMC1205530 8349118

[B70] Rozas J Sanchez-Del Rio JC Messeguer X Rozas R DnaSP, DNA polymorphism analyses by the coalescence and other methods Bioinformat 2003 19 2496 2497 10.1093/bioinformatics/btg359 14668244

[B71] Tajima F Statistical-method for testing the neutral mutation hypothesis by DNA polymorphism Genet 1989 123 585 595 10.1093/genetics/123.3.585 PMC1203831 2513255

[B72] Fu YX Li WH Statistical tests of neutrality of mutations Genet 1993 133 693 709 10.1093/genetics/133.3.693 PMC1205353 8454210

[B73] Ramos-Onsins SE Rozas J Statistical properties of new neutrality tests against population growth Mol Biol Evol 2002 19 2092 2100 12446801 10.1093/oxfordjournals.molbev.a004034

[B74] Fu YX Statistical tests of neutrality of mutations Genet 1997 133 693 709 10.1093/genetics/133.3.693 PMC1205353 8454210

[B75] Kumar S Tamura K Nei M MEGA3: Integrated software for Molecular Evolutionary Genetics Analysis and sequence alignment Brief Bioinform 2004 5 150 163 10.1093/bib/5.2.150 15260895

[B76] Crawford MH Molecular Markers in Anthropological Genetic Studies Anthropological genetics: theories, methods and applications 2007 Cambridge University Press 141 186

[B77] Harpending HC Signature of ancient population growth in a low-resolution mitochondrial DNA mismatch distribution Human Biol 1994 66 591 600 8088750

[B78] Zarza E Reynoso VH Emerson BC Diversification in the northern neotropics: mitochondrial and nuclear DNA phylogeography of the iguana *Ctenosaura pectinata *and related species Mol Ecol 2008 17 3259 3275 10.1111/j.1365-294X.2008.03826.x 18564087

[B79] Linthicum KJ A revision of the Argyritarsis Section of the subgenus *Nyssorhynchus *of *Anopheles *(Diptera: Culicidae) Mosq Sys 1988 20 98 271

[B80] Kimura M Crow JF The number of alleles that can be maintained in a finite population Genet 1964 49 725 38 10.1093/genetics/49.4.725 PMC1210609 14156929

[B81] Brower A Rapid morphological radiation and convergence among races of the butterfly *Heliconius erato *inferred from patterns of mitochondrial DNA evolution Proc Natl Acad Sci USA 1994 91 6491 6495 10.1073/pnas.91.14.6491 8022810 PMC44228

[B82] Reisz RR Muller J Molecular timescales and the fossil record: a paleontological perspective Trends Genet 2004 20 237 241 10.1016/j.tig.2004.03.007 15109777

[B83] Krzywinski J Besansky N Molecular systematics of *Anopheles*: from subgenera to subpopulations Annu Rev Entomol 2003 48 111 139 10.1146/annurev.ento.48.091801.112647 12208816

[B84] Slatkin M Hudson RR Pairwise comparisons of mitochondrial DNA sequences in stable and exponentially growing populations Genet 1991 129 55 562 10.1093/genetics/129.2.555 PMC1204643 1743491

[B85] Loaiza J Scott M Bermingham E Rovira J Conn JE Evidence for Pleistocene population divergence and expansion of *Anopheles albimanus *in southern Central America Am J Trop Med Hyg 2010 82 156 164 10.4269/ajtmh.2010.09-0423 20065014 PMC2803528

[B86] Akey JM Eberle MA Rieder MJ Carlson CS Shriver MD Nickerson DA Kruglyak L Population history and natural selection shape patterns of genetic variation in 132 genes PLoS Biol 2004 2 1591 1599 10.1371/journal.pbio.0020286 PMC515367 15361935

[B87] Harpending HC Sherry ST Rogers AR Stoneking M The genetic structure of ancient human populations Curr Anthropol 1993 34 483 496 10.1086/204195

[B88] Hasan AU Suguri S Fujimoto C Londari Itaki R Masakazu Harada Kawabata M Bugoro H Albino B Tsukahara T Hombhanje F Masta A Phylogeography and dispersion pattern of *Anopheles farauti senso stricto *mosquitoes in Melanesia Mol Phylogenet Evol 2008 46 792 800 10.1016/j.ympev.2007.09.018 18357645

[B89] Zink RM Barrowclough GF Mitochondrial DNA under siege in avian phylgeography Mol Ecol 2008 17 2107 2121 10.1111/j.1365-294X.2008.03737.x 18397219

[B90] Hellberg ME No variation and low synonymous substitution rates in coral mtDNA despite high nuclear variation BMC Evol Biol 2006 6 10.1186/1471-2148-6-24 16542456 PMC1431588

[B91] He L Watabe H Xiangyu J Gao J Liang XC Aotsuka T Zhang Y Genetic differentiation and cryptic speciation in natural populations of *Drosophila lacertosa* Mol Phylogenet and Evol 2007 43 24 31 10.1016/j.ympev.2006.08.010 17011795

[B92] Crywinska A Hunter FF Hebert PDN Identifying Canadian mosquito species through DNA barcodes Med Vet Entomol 2006 20 413 424 10.1111/j.1365-2915.2006.00653.x 17199753

[B93] Kumar NP Rajavel AR Natarajan R Jambulingam P DNA Barcodes Can Distinguish Species of Indian Mosquitoes (Diptera: Culicidae) J Med Entomol 2007 44 1 7 10.1603/0022-2585(2007)44[1:DBCDSO]2.0.CO;2 17294914

[B94] Arrivillaga JC Norris DE Feliciangeli MD Lanzaro GC Phylogeography of the neotropical sand fly *Lutzomyia longipalpis *inferred from mitochondrial DNA sequences Infect Genet Evol 2002 2 83 95 10.1016/S1567-1348(02)00087-4 12797984

[B95] Monteiro FA Barrett TV Fitzpatrick S Cordon-Rosales C Feliciangeli D Beard CB Molecular phylogeography of the Amazonian Chagas disease vectors *Rhodnius prolixus *and *R. robustus* Mol Ecol 2003 12 997 1006 10.1046/j.1365-294X.2003.01802.x 12753218

[B96] Kaandorp RJG Vonhof HB Wesselingh FP Pittman LR Kroon D van Hinte JE Seasonal Amazonian rainfall variation in the Miocene Climate Optimum Palaeogeogr Palaeoclimatol Palaeoecol 2005 221 1 6 10.1016/j.palaeo.2004.12.024

[B97] Yu Y Huber M Muller RD Poulsen CJ Ribbe J Simulation of the Middle Miocene Climate Optimum Geophys Res Lett 2009 36 L04702 10.1029/2008GL036571

[B98] Bonaccorso E Kock I Peterson AT Pleistocene fragmentation of Amazon species' ranges Divers Distrib 2006 12 157 164 10.1111/j.1366-9516.2005.00212.x

[B99] Mayle FE Beerling DJ Gosling WD Bush MB Responses of Amazonian ecosystems to climatic and atmospheric carbon dioxide changes since the last glacial maximum Philos Trans R Soc Lond B Biol Sci 2004 359 499 514 10.1098/rstb.2003.1434 15212099 PMC1693334

[B100] Kress WJ Heyer WR Acevedo P Coddington J Cole D Erwin Tl Meggers BJ Pogue M Thorington RW Vari RP Weitzman MJ Weitzman SH Amazonian biodiversity: assessing conservation priorities with taxonomic data Biodivers Conserv 1998 7 1577 1587 10.1023/A:1008889803319

[B101] Pedro PM Sallum MA Spatial expansion and population structure of the neotropical malaria vector, *Anopheles darlingi *(Diptera: Culicidae) Biol J Linn Soc 2009 97 854 866 10.1111/j.1095-8312.2009.01226.x

[B102] Conn JE Mitchell SE Cockburn AF Mitochondrial DNA analysis of the neotropical malaria vector *Anopheles nuneztovari* Genome 41 313 327 10.1139/gen-41-3-313

[B103] Solomon SE Bacci M Jr Martins J Jr Vinha GG Mueller UG Paleodistributions and comparative molecular phylogeography of leafcutter ants (*Atta *spp.) provide new insight into the origins of Amazonian diversity PLoS ONE 2008 3 10.1371/journal.pone.0002738 18648512 PMC2447876

[B104] Hawkins BA Field R Cornell HV Currie DJ Guegan JF Jaufman DM Kerr JT Mittelbach GG Oberdorff T O'Brien EM Porter EE Turner JRG Energy, water, and broad-scale geographic patterns of species richness Ecol 2003 84 3105 3117 10.1890/03-8006

[B105] Vasconcelos HL Vilhena JMS Facure KG Albernaz ALKM Patterns of ant species diversity across 2000 km of Amazonian floodplain forest J Biogeog 2009 37 432 440 10.1111/j.1365-2699.2009.02230.x

[B106] Sombroek W Spatial and temporal patterns of Amazon rainfall: consequences for the planning of agricultural occupation and the protection of primary forests AMBIO: J Human Environ 2001 30 388 396 10.1579/0044-7447-30.7.388 11795213

[B107] Maeglin RR Forest products from Latin America: an almanac of the state of the knowledge and the state of the art. United States Forest Service, Dept of Agriculture, Gen Tech Report 1991 FPL-GTR-67

[B108] Absy ML Cleef A Fournier M Servant M Siffedine A Silva MFF Suguio K Turcq B Van der Hammen T Mise en évidence de quatre phases d'ouverture de la forêt dense dans le sud-est de l'Amazonie au cours des 6000 dernières années Première comparaison avec d'autres régions tropicales. Compte Rendus de l'Académie des sciences 2nd series 1991 312 673 678

[B109] Rull V Holocene global warming and the origin of the Neotropical Gran Sabana in the Venezuelan Guayana J Biogeog 2007 34 279 88 10.1111/j.1365-2699.2006.01620.x

[B110] Mayle FE Power MJ Impact of a drier Early-Mid-Holocene climate upon Amazonian forests Philos Trans R Soc Lond Biol Sci 2008 363 829 38 10.1098/rstb.2007.0019 PMC2374889 18267912

[B111] Pasteur N Raymond M Insecticide resistance genes in mosquitoes: Their mutations, migration, and selection on field populations J Hered 1996 87 444 449 8981762 10.1093/oxfordjournals.jhered.a023035

[B112] Akhavan D Musgrove P Abrantes A Gusmao RD'A Cost-effective malaria control in Brazil. Cost-effectiveness of a malaria control program on the Amazon Basin of Brazil, 1988-96 Soc Sci Med 1999 49 1385 1399 10.1016/S0277-9536(99)00214-2 10509828

[B113] Smith HM The perspective of species Turtox News 1955 33 74 77

[B114] Smith HM More evolutionary terms J Syst Zool 1965 14 57 58 10.2307/2411904

[B115] Schnabel KE Hebert PDN Resource-associated divergence in the arctic marine amphipod *Paramphithoe hystrix* Marine Biol 2003 143 851 857 10.1007/s00227-003-1126-4

[B116] Friesen Vl Smith AL Gómez-Díaz E Bolton M Furness RW González-Solís J Monteiro LR Sympatric speciation by allochrony in a seabird Proc Natl Acad Sci USA 2007 104 18589 18594 10.1073/pnas.0700446104 18006662 PMC2141821

