# Supplementary figures and images for: Lineage divergence detected in the malaria vector Anopheles marajoara (Diptera: Culicidae) in Amazonian Brazil
Source: Malar J. 2010 Oct 7;9:271. doi: 10.1186/1475-2875-9-271 (PMC2959070; doi:10.1186/1475-2875-9-271)

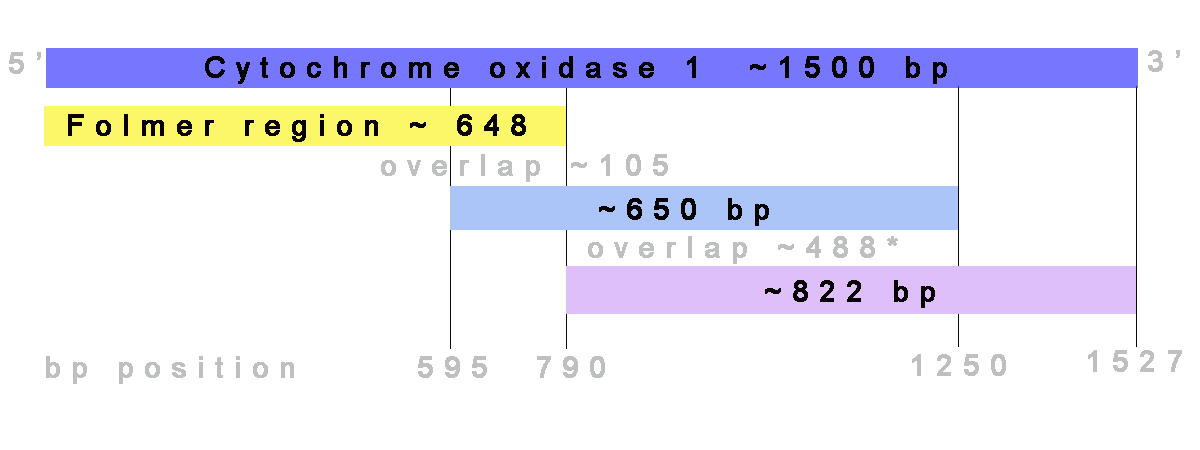

Supplement: Additional file 1 — Complete COI with fragment orientation and overlap. Representative schematic of the COI gene, Folmer region and fragments; light blue bar indicating the region amplified by primers UEA3 and UEA10; the purple bar depicting fragment previously amplified from primers 2195D and C1-J-2195. *, denotes the piece of the COI that was used for the phylogeography and population structure analysis. [file 1475-2875-9-271-S1.PNG]
